# Supplementary material for: Alteration of adeS Contributes to Tigecycline Resistance and Collateral Sensitivity to Sulbactam in Acinetobacter baumannii
Source: Microbiol Spectr. 2023 May 15;11(3):e04594-22. doi: 10.1128/spectrum.04594-22 (PMC10269438; doi:10.1128/spectrum.04594-22)
Supplement: Supplemental file 1 — Supplemental material. Download spectrum.04594-22-s0001.pdf, PDF file, 0.6 MB [file spectrum.04594-22-s0001.pdf]

Table S1. The primers used in the study.

Primers	Sequence
rpoB-RT-F	5'-TCCGCACGTAAAGTAGGAAC-3′
rpoB-RT-R	5'-ATGCCGCCTGAAAAAGTAAC-3′
OXA-23-RT-F	5'-TATTCTGTATTTGCGCGG-3′
OXA-23-RT-R	5'-CT ATGTGGTTGCTTCTCT-3'
adeB-RT-F	5′-AATGGAATAAGGCACCACAACA-3′
adeB-RT-R	5′-ACCAAAGGAATTACACCACACG-3′
